# Image quality and clinical usefulness of automatic tube current modulation technology in female chest computed tomography screening

**DOI:** 10.1097/MD.0000000000021719

**Published:** 2020-08-14

**Authors:** Cheng Li, Lin Qi, Yusheng Zhang, Feng Gao, Xiu Jin, Lukai Zhang, Huan Tang, Ming Li

**Affiliations:** a Department of Radiology, Huadong Hospital Affiliated to Fudan University; b Institute of Functional and Molecular Medical Imaging, Fudan University, Shanghai, China.

**Keywords:** breast, computed tomography, dose-saving technique, radiation, X-ray combined applications to reduce exposure

## Abstract

The study was aimed to evaluate the image quality and radiation dose during female chest computed tomography (CT) screening using organ-based tube current modulation technology X-ray combined applications to reduce exposure (XCARE).

Five hundred sixty female individuals undergoing chest CT scan were prospectively enrolled and divided into 4 groups based on body mass index (BMI). Then they were randomly and equally divided into control and experimental subgroup and respectively accepted conventional low-dose and XCARE technology spiral CT scan with same parameters. Signal-to-noise ratio and contrast-to-noise ratio were calculated. The quality of the images was evaluated by 2 radiologists using a 5-point scale.

Among experimental subgroups of the 4 BMI groups, Signal-to-noise ratios, CT dose index of volume, dose-length product, effective dose, and contrast-to-noise ratio all displayed significant differences, as well as in control subgroups (*P* < .001). Both the experimental and control subgroups showed an increasing trend in radiation dose with the increasing of BMI. Parameters of image quality and radiation dose displayed no significant differences between control and experimental subgroups in the 4 groups. In multiple linear regression analysis, age and scanning protocol were not associated with radiation dose (*P* *>* .05), while BMI was significantly associated with increased CT dose index of volume (*P* < .05). The display of the lesions for the patients in the control and experimental subgroups of the 4 groups with different BMIs exhibited no statistically significant difference.

The same image quality and radiation dose can be obtained using XCARE technology compared to conventional chest CT scans, which can be used regularly in female patients.

Advances in knowledge: Using automatic tube current modulation technology to reduce exposure in breast. In this study, we sought a radiation protection method for sensitive tissue in chest CT screening.

## Abstract 2

Supplemental Digital Content is available in the text

## 1 Introduction

Currently, low-dose helical computed tomography (CT) screening for lung cancer has been widely accepted.^[1,2]^ However, with conventional chest CT scans, the mammary gland is unavoidably exposed to direct radiation. Recently research shows that digital mammography average glandular dose is the same range as glandular tissue dose for lung cancer screening CT exams.^[3]^ The mammary gland is highly sensitive to radiation.^[4]^ Therefore, reducing direct radiation of the breast is extremely important for the prevention of breast cancer and breast diseases in women who undergo a CT scan during a physical or follow-up examination.

The X-ray combined applications to reduce exposure (XCARE) scan sequence is option of Siemens application. XCARE uses exposure technology that involves an automatically adjusted tube current. That is, when the tube is rotated to the position above the body, the tube current is reduced to lower the radiation dose to the sensitive area, and when the tube is rotated to the back side of the body, the tube current is adjusted back to the normal level. The lowest tube current is reduced to 25% of the average, which can effectively reduce the radiation dosage to the sensitive area of patient.^[5]^

This study aimed to apply the XCARE technology to female CT screening for lung disease and to explore the application value of the XCARE technology in female lung screening compared with regular chest CT scan.

## 2 Patients and methods

This study was approved by the Institutional Review Board of our hospital and informed consent was sought from all individuals participating in the study.

### 2.1 Patient selection

**Figure 1:**
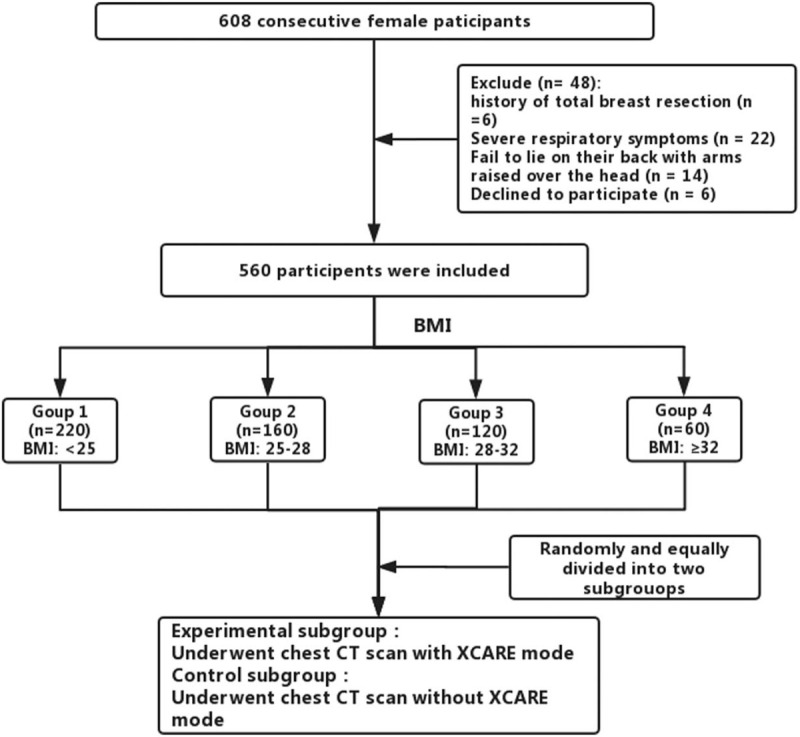
Flowchart of research participants examined by chest CT scan. BMI = body mass index, CT = computed tomography.

From February 2018 to August 2018, we prospectively assessed 608 consecutive female participants referred to our department for chest CT screening. Exclusion criteria included history of total breast resection (n = 6), severe respiratory symptoms that interfered with the process of CT scan (n = 22), fail to lie on their back with arms raised over the head (n = 14), declined to participate (n = 6). Finally, a total of 560 patients were enrolled in our study. Patient height and weight were recorded before CT scan to calculate body mass index (BMI = body weight/height^2^ [kg/m^2^]). All the participants were divided into the following 4 groups based on BMI: Group 1, BMI <25; Group 2, BMI ≥25 and <28; Group 3, BMI ≥28 and <32; Group 4, BMI ≥32. Then each group of participants were randomly and equally divided into experimental and control subgroup (Fig. 1).

### 2.2 Chest CT scan protocol

All participants were scanned with a Somatom Definition Flash CT (Siemens Healthineers, Germany) instrument, and the scan range covered the entire lung, from the level of the lung apex to the liver dome. Patients were required to hold their breath at the end of deep inhalation during the examination. For all the participants, the underwear and other metal objects were removed before the scanning procedure.

**Figure 2:**
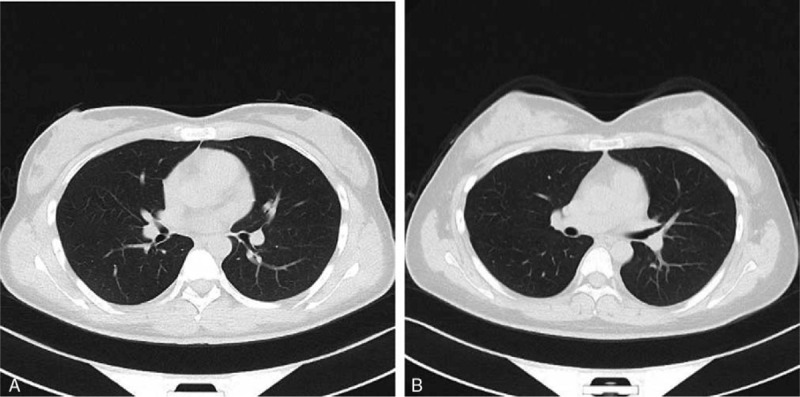
The quality of all the images met the diagnostic requirements. (A) Displays a case in which the XCARE technology was not used, while (B) shows a case with the use of the XCARE technology. XCARE = X ray combined applications to reduce exposure.

The scanning parameters were as follows: for the experimental group, 100 kV and CAREDose4D, with 5-mm and 1-mm layer thickness; the collimator was 128 × 0.6 mm; pitch 0.6; option value of sinogram affirmed iterative reconstruction (SAFIRE) was 2; the convolution kernel of the pulmonary window I70f was very sharp; the mediastinal window of I31 was medium smooth; none IV contrast and the scanning program that included the XCARE function to protect sensitive organs was selected for the scanning procedure.^[6]^ For the control group, all the conditions were identical to those for the experimental group except that the XCARE function was not used (Fig. 2).

### 2.3 Image quality assessment

The 5-mm layer thickness axial images were selected for each patient. The left pectoralis major on the apex level, the descending aorta on the carina level, and the left atrium were selected in the mediastinal window (width, 400 HU; level, 40 HU) as the region of interest to measure and record the average CT HU value and background noise (standard deviation [SD]). The circular region of interest was 200 mm^2^. Signal-to-noise ratio (SNR) and contrast-to-noise ratio (CNR) were calculated. CNR = (average CT HU value of descending aorta – average CT HU value of same layer muscle)/background noise.^[7]^

All patent and scanner demographic data were removed and the analysis of images was independently performed by 2 senior radiologists with a double-blind method. The clarity of the display of the mediastinum, great vessels, pleura, chest wall soft tissue, lung segment, and sub-segmental bronchi, as well as the details of anatomical structures, particle size, and artifacts were primarily observed. A 5-point scale was used as the criteria to subjectively assess the image quality^[4]^: 5, anatomic details and lesions were clearly shown, and the image could be simply and clearly evaluated; 4, the anatomical structures and details as well as the lesions were somewhat clearly shown, and the image was evaluable but was not particularly good; 3, most of the anatomical structures and lesions could meet the diagnostic requirements, but a small number of images could not be evaluated; 2, the display of the anatomical structures and lesions was not clear, and the details could not be observed; 1, the anatomical structures and lesions were too vague for a diagnosis. A score of 3 and above can meet the diagnostic requirements.

The anatomical details were associated with the analysis of the mediastinal window and lung window (width, 1500 HU; level, –700 HU), including the ability to display the lesion morphology, size, and boundary. The lesions (normal, ground-glass nodules, fibrous cord, calcification, empty cavity, and lesion of thymus) in each patient were evaluated by 2 radiologists.

### 2.4 Radiation dose

The CT dose index of volume (CTDI_vol_) and dose-length product (DLP) for each participant were recorded. The CTDI_vol_ reflects the average dose throughout the entire scan volume, while the DLP was used to evaluate the total radiation dose for the participant during a full CT scan.^[8,9]^ DLP was converted to effective dose (ED) in millisieverts (mSv) by multiplying it by the thoracic conversion factor of 0.0144 mSv mGy^−1^cm^−1^.^[10]^

### 2.5 Statistical analysis

Statistical analysis was carried out by SPSS 22.0 software (IBM SPSS Statistics, Armonk, NY) and GraphPad Prism (Prism for Windows, Version 7.0a; Graph-Pad Software, Inc., La Jolla, CA). Values were described with either mean ± SD or median with interquartile range after testing the normality of variables using Shapiro–Wilk test. Parameters among the 4 groups were compared by Kruskal–Walls test. The average CT HU value, the noise (SD), SNR, CNR, CTDI_vol_, and DLP for the 2 subgroups of images were compared with the Mann–Whitney *U* test or Dunnett *t*-test for 2 independent samples. The agreement of the scores obtained from the evaluation by different physicians was determined using the *Kappa* test, for which can be interpreted as follows: ≤0, no agreement; 0.01 to 0.20, as none to slight; 0.21 to 0.40, as fair; 0.41 to 0.60, as moderate; 0.61 to 0.80, as substantial; 0.81 to 1.00, as almost perfect agreement. Multiple regression analysis was used to determine the independent predictors of radiation dose (CTDI_vol_), and Pearson correlation analysis was performed between the predictor and radiation dose. A *P*-value of less than .05 was considered significant.

## 3 Results

### 3.1 Descriptive statistics and image quality in 4 groups

Descriptive statistics of the parameters of 4 groups were summarized in Table 1. All the 560 female participants successfully completed the chest CT scan (age, 48 ± 15.0 years; BMI, 26.2 ± 4.2 kg/cm^2^). The ED was 2.4 ± 0.8 mSv. No significant difference was found with respect to age among the 4 groups. But CTDI_vol_, DLP, ED, SNRs, and CNR were significantly different among them (*P* < .001). CTDI_vol_, DLP, and ED showed an increasing trend with the increase of BMI.

**Table 1:**
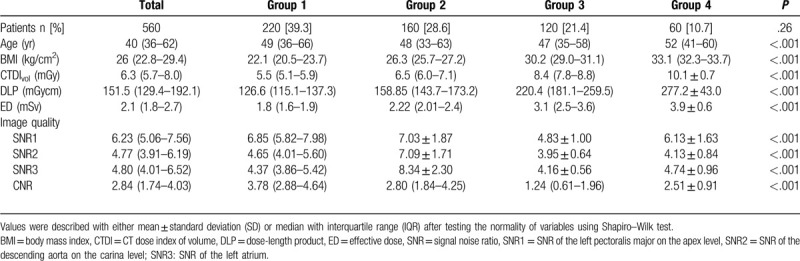
Patient characteristics, radiation dose, and image quality in the 4 groups.

**Figure 3:**
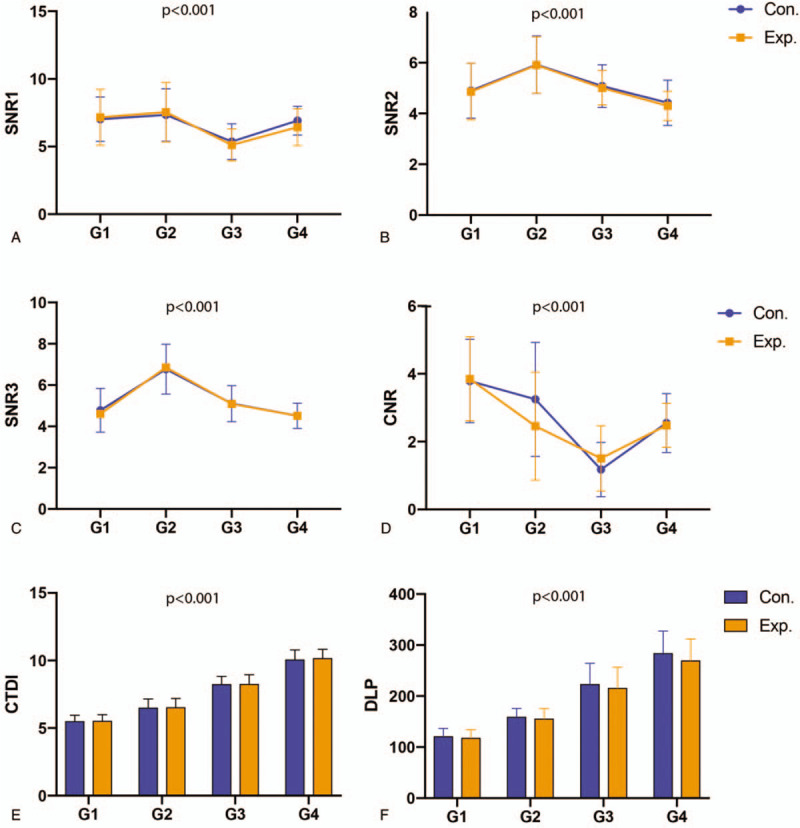
Among experimental subgroups of the 4 BMI groups, SNRs at different levels, CTDI_vol_, DLP, ED, and CNR all displayed significant differences, as well as that in control subgroups. Both the experimental and the control subgroup obtained the maximum SNR value in group 2 (A–C), and the maximum CNR value in group 1 (BMI <25). In control subgroup, both of the BMI <25 and BMI 25 to 28 groups could obtain higher CNR values, while in experimental subgroup, there was no advantage of CNR between BMI 25 to 28 (group 2) and BMI >32 (group 4) (D). Both the experimental and control subgroups showed an increasing trend in radiation dose with the increasing of BMI (*P* > .001) (E and F). BMI = body mass index, CTDI = CT dose index of volume, DLP = dose-length product, SNR = signal noise ratio, SNR1 = SNR of the left pectoralis major on the apex level, SNR2 = SNR of the descending aorta on the carina level; SNR3: SNR of the left atrium.

Among experimental subgroups of the 4 BMI groups, SNRs at different levels, CTDI_vol_, DLP, ED, and CNR all displayed significant differences, as well as that in control subgroups (*P* < .001). Both the experimental and the control subgroup obtained the maximum SNR value in group 2 (BMI, 25–28) (Fig. 3A–C), and the maximum CNR value in group1 (BMI <25). In control subgroup, both of the BMI <25 and BMI 25 to 28 groups could obtain higher CNR values, while in experimental subgroup, there was no advantage of CNR between BMI 25 to 28 (group 2) and BMI >32 (group 4) (Fig. 3D). Both the experimental and control subgroups showed an increasing trend in radiation dose with the increasing of BMI (*P* > .001) (Fig. 3E and F).

### 3.2 Image quality and radiation dose in subgroups

Table 2 summarizes the average CT values, the noise, and SNR at different levels, as well as CNR in control and experimental subgroups at different BMI levels, which were not significantly different from each other. The radiation doses including CDTI_vol_ and DLP for the subgroups were shown in Table 3. There were no significant differences between control and experimental subgroups in 4 groups.

**Table 2:**
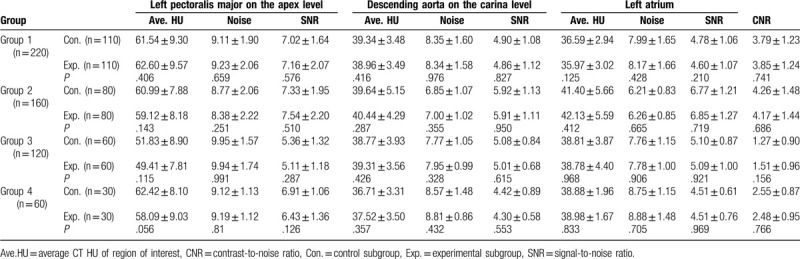
Comparison of the average CT values, the noise (standard deviation, SD), the signal to noise ratio (SNR) and contrast to noise ratio (CNR) between control subgroup and experimental subgroup in the 4 groups.

**Table 3:**
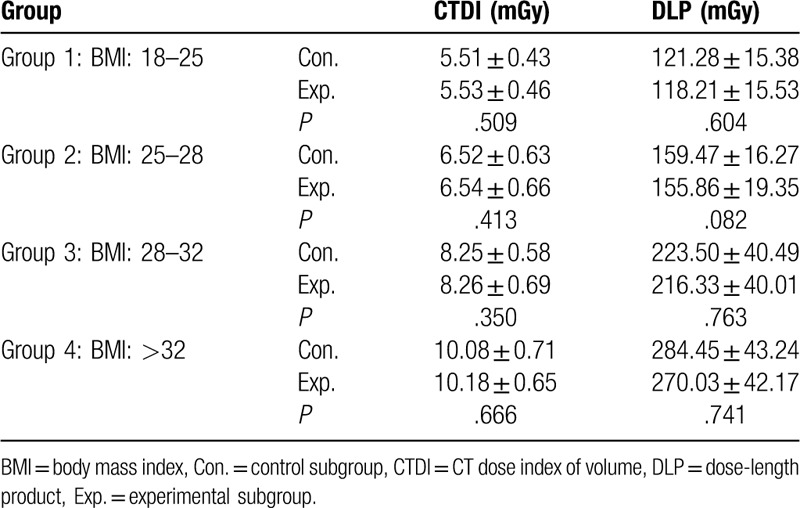
Comparison of the radiation dose between the subgroups.

The scores reflecting the image quality for the control and experimental subgroups in the 4 groups with different BMIs were showed in Table 4, and none of differences between the experimental and control subgroups were statistically significant (*P* > .05). Good inter-observer agreement was found for Group 1, and moderate inter-observer agreement was found for Group 2, 3, and 4. Furthermore, the quality of all the images met the diagnostic requirements (Fig. 2). In multiple linear regression analysis, age (*β* = 0.001, 95% confidence interval, −0.01 to 0.00, *P* *>* .05) and scanning protocol (*β* = 0.02, 95% confidence interval, −0.12 to 0.15, *P* *>* .05) were not associated with radiation dose, while BMI was significantly associated with increased CTDI_vol_ (*β* = 0.34, 95% confidence interval, 0.32–0.35, *P* < 0.05) (Supplement Table). Correlation was significant between BMI and CTDI_vol_ (correlation coefficient, 0.863, *P* = .000).

**Table 4:**
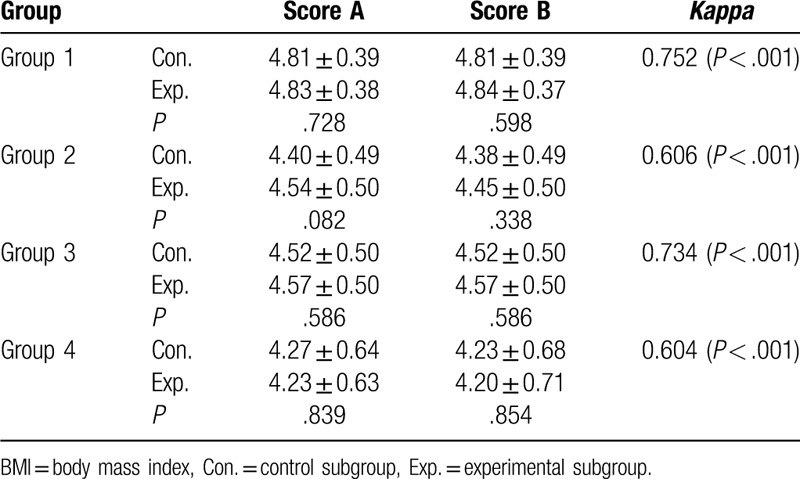
Comparison of the subjective rating for the images between the 2 subgroups, the control group and the experimental group, in the 4 BMI groups. The inter-observer consistency for each BMI subgroup (control/experimental group in total) is listed in the right column.

### 3.3 Number of detected lesions

The display of the lesions for the patients in the control and experimental subgroups of the 4 groups with different BMIs exhibited no statistically significant difference (Table 5). In the control group, 3 cases were diagnosed with ground glass lesions of 3 to 6 mm, in 2 cases of which the lesions were in the right upper lung lobe, and the lesion was in the left lung lobe in 1 case. In the experimental group, 4 cases were diagnosed with ground glass lesions of 3 to 5 mm; in 2 cases, the lesions were in the right upper lobe, and in 1 case each, the lesion was in the right middle lobe and the left upper lobe.

**Table 5:**
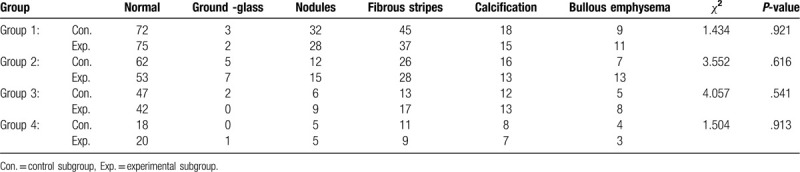
Display of lesions in the images for the 2 subgroups, the control group and the experimental group, in the 4 groups.

## 4 Discussion

With consideration of the extremely radiosensitive glandular tissue in the female breast, dose saving algorithms have to be applied, if possible. In chest CT examinations, breasts are usually not diagnostically targeted, but they receive a considerable amount of unnecessary radiation dose.^[11]^ Previous studies have shown that the tissues in the mammary gland are highly sensitive to radiation, and the irradiation of these tissues is thus more likely to cause tumors than the irradiation of other organs.^[12,13]^ One of the main goals is to minimize the exposure of the breast to radiation. The increasing number of diagnosed breast cancer cases is a cause of concern. A number of studies evaluated the influence of medical radiation exposure on future occurrence of malignant tumors in women who were exposed to high radiation doses due to multiple radiation examinations or treatment by radiotherapy.^[14–16]^ However, the advantages of lung CT examination in revealing small pulmonary lesions has gradually become an important tool in physical examinations. In the conventional chest CT scanning procedure, the direct exposure of the breast to radiation is not avoidable, and the breast is not the target organ in lung CT screening; therefore, it is self-evident that the biological effect of frequent examinations or large doses of radiation might be a potential cancer risk.^[13]^

Automatic tube-current modulation techniques have been widely used in CT examination.^[17]^ Spiral CT, as the name suggests, is the mode in which the tube and the detector rotate around the body, and the X-ray emitted from the tube arrives at the receiving detector through the body to obtain the image information after photoelectric conversion. Some vendors have developed organ-based tube current modulation techniques to protect superficial radiosensitive organs, such as breasts. It has been reported that with the application of organ-based tube current modulation (X-CARE, Siemens Healthcare), the tube current (mA) is reduced by 80% with a corresponding increase in the posterior region, thereby reducing the risk of breast diseases.^[5,18,19]^ Lungren MP et al^[20]^ reported that, with organ-based tube current modulation in adult anthropomorphic phantom, breast dose can be reduced by 17% to 47% with no detrimental effect on image quality. Wanyi Fu et al^[16]^ reported that organ-based tube current modulation reduced breast dose by 38.6% ± 8.1% in 13 female anthropomorphic computational phantoms. However, there is still a lack of clinical research on the application of organ-based tube current modulation technology in female patients. Similar to the results of previous phantom researches, in our study, the objective parameters and subjective evaluation of the image quality, as well as the radiation dose for the experimental group after using the XCARE technology revealed no significant difference compared with the control group. Because the chest CT scan protocol automatically adjusts the tube voltage based on the patient's BMI, this study was based on a BMI stratification analysis and we concluded that there were no statistically significant differences in image quality and radiation dose between the experimental and control groups within the same BMI range.

Thus, the tube current is reduced for tube position, where x-rays pass the patient from anterior to posterior, to reduce the direct exposure of radiosensitive organs. To maintain image quality, the tube current is increased for the remaining projections (posterior to anterior) to get the same milliampere per rotation, the shaded area indicates the region for very low-dose X-ray emission and the tube current is decreased. Considering the radiation doses distributed non-uniformly in the scan region, exposure to breast areas remain low compared to other areas of the body.

It indicated that the application of the XCARE technology for lung CT screening could reduce the irradiation of the breast while maintaining the same image quality in the lung CT scan. In the subjective rating, the display of the lung structure and lesions also showed no difference between the 2 groups of images, and the images of both groups could reveal the lesions well. Both the control and experimental groups exhibited a good performance in the detection and details of small lesions, with clear anatomical structures of the lesions, including fibrous stripes, calcification, and bullous emphysema, and the difference was not statistically significant, thus meeting the diagnostic requirements.

One of the limitations of our study is that no specific instrument is used to measure the radiation dose received by the patient's mammary gland during the examination. In summary, the XCARE technology in female chest CT screening can obtain the same image quality and radiation dose compared to conventional chest CT scans, which can be used regularly in female patients.

## Author contributions

**Conceptualization:** Cheng Li.

**Data curation:** Cheng Li.

**Investigation:** Yusheng Zhang, Lukai Zhang.

**Methodology:** Lin Qi.

**Resources:** Feng Gao.

**Software:** Feng Gao, Lukai Zhang.

**Supervision:** Xiu Jin.

**Validation:** Xiu Jin.

**Visualization:** Huan Tang.

**Writing – original draft:** Cheng Li, Ming Li.

**Writing – review & editing:** Cheng Li, Lin Qi.

## Supplementary Material

Supplemental Digital Content

## References

[1] Blanchon T Brechot JM Grenier PA Baseline results of the Depiscan study: a French randomized pilot trial of lung cancer screening comparing low dose CT scan (LDCT) and chest X-ray (CXR). Lung Cancer (Amsterdam, Netherlands) 2007;58:50–8. 10.1016/j.lungcan.2007.05.009 17624475

[2] Borondy Kitts AK The patient perspective on lung cancer screening and health disparities. J Am Coll Radiol 2019;16:601–6. 30947894 10.1016/j.jacr.2018.12.028

[3] Hardy AJ Bostani M McMillan K Estimating lung, breast, and effective dose from low-dose lung cancer screening CT exams with tube current modulation across a range of patient sizes. Med Physics 2018;45:4667–82. 10.1002/mp.13131 PMC6220713 30118143

[4] Biermann J Langen B Nemes S Radiation-induced genomic instability in breast carcinomas of the Swedish hemangioma cohort. Genes Chromosomes Cancer 2019;58:627–35. 30938900 10.1002/gcc.22757

[5] Ketelsen D Buchgeister M Fenchel M Automated computed tomography dose-saving algorithm to protect radiosensitive tissues: estimation of radiation exposure and image quality considerations. Invest Radiol 2012;47:148–52. 21934513 10.1097/RLI.0b013e3182311504

[6] Yang WJ Yan FH Liu B Can sinogram-affirmed iterative (SAFIRE) reconstruction improve imaging quality on low-dose lung CT screening compared with traditional filtered back projection (FBP) reconstruction? J Comput Assist Tomogr 2013;37:301–5. 23493224 10.1097/RCT.0b013e31827b8c66

[7] Tang H Yu N Jia Y Assessment of noise reduction potential and image quality improvement of a new generation adaptive statistical iterative reconstruction (ASIR-V) in chest CT. Br J Radiol 2018;91:20170521. 29076347 10.1259/bjr.20170521 PMC5966217

[8] Hidajat N Maurer J Schroder RJ Relationships between physical dose quantities and patient dose in CT. Br J Radiol 1999;72:556–61. 10560337 10.1259/bjr.72.858.10560337

[9] Liu W Ding X Kong B Reducing the radiation dose with the adaptive statistical iterative reconstruction technique for chest CT in adults: a parameter study. Chin Med J 2014;127:1284–8. 24709181

[10] Deak PD Smal Y Kalender WA Multisection CT protocols: sex- and age-specific conversion factors used to determine effective dose from dose-length product. Radiology 2010;257:158–66. 20851940 10.1148/radiol.10100047

[11] Barcellos-Hoff MH New biological insights on the link between radiation exposure and breast cancer risk. J Mammary Gland Biol Neoplasia 2013;18:3–13. 23325014 10.1007/s10911-013-9272-x

[12] Martin K Vogel RI Nagler RH Mammography Screening Practices in Average-Risk Women Aged 40-49 Years in Primary Care: A Comparison of Physician and Nonphysician Providers in Minnesota. J Womens Health (Larchmt) 2020;29:91–9. 31314684 10.1089/jwh.2018.7436 PMC6983752

[13] Kidoh M Utsunomiya D Oda S Breast dose reduction for chest CT by modifying the scanning parameters based on the pre-scan size-specific dose estimate (SSDE). Eur Radiol 2017;27:2267–74. 27718079 10.1007/s00330-016-4618-6

[14] Franck C Smeets P Lapeire L Estimating the patient-specific dose to the thyroid and breasts and overall risk in chest CT when using organ-based tube current modulation. Radiology 2018;288:164–9. 29584596 10.1148/radiol.2018170757

[15] Vollmar SV Kalender WA Reduction of dose to the female breast in thoracic CT: a comparison of standard-protocol, bismuth-shielded, partial and tube-current-modulated CT examinations. Eur Radiol 2008;18:1674–82. 18414873 10.1007/s00330-008-0934-9

[16] Fu W Tian X Sturgeon GM CT breast dose reduction with the use of breast positioning and organ-based tube current modulation. Med Physics 2017;44:665–78. 10.1002/mp.12076 28032894

[17] Spampinato S Gueli AM Milone P Dosimetric changes with computed tomography automatic tube-current modulation techniques. Radiol Physics Technol 2018;11:184–91. 10.1007/s12194-018-0454-5 29626289

[18] Muller K Meineke V Advances in the management of localized radiation injuries. Health Physics 2010;98:843–50. 20445392 10.1097/HP.0b013e3181adcba7

[19] Wang J Duan X Christner JA Radiation dose reduction to the breast in thoracic CT: comparison of bismuth shielding, organ-based tube current modulation, and use of a globally decreased tube current. Med Physics 2011;38:6084–92. 10.1118/1.3651489 22047373

[20] Lungren MP Yoshizumi TT Brady SM Radiation dose estimations to the thorax using organ-based dose modulation. AJR Am J Roentgenol 2012;199:W65–73. 22733933 10.2214/AJR.11.7798

