# Supplementary material for: Image quality and clinical usefulness of automatic tube current modulation technology in female chest computed tomography screening
Source: Medicine (Baltimore). 2020 Aug 14;99(33):e21719. doi: 10.1097/MD.0000000000021719 (PMC7437791; doi:10.1097/MD.0000000000021719)
Supplement: Supplemental Digital Content [file medi-99-e21719-s001.docx]

Supplement Table: Multiple linear regression analysis the independent predictors of radiation dose (CTDI_vol_).

|  | β value | 95% CI | p value |
| --- | --- | --- | --- |
| Age | 0.001 | -0.01-0.00 | >0.05 |
| Scanning Protocol | 0.02 | -0.12-0.15 | >0.05 |
| BMI | 0.34 | -0.12-0.15 | <0.05 |

BMI=body mass index; CI=confidence interval.
